# PPAR*γ* and Agonists against Cancer: Rational Design of Complementation Treatments

**DOI:** 10.1155/2008/945275

**Published:** 2008-11-18

**Authors:** Dorina Veliceasa, Frank Thilo Schulze-Hoëpfner, Olga V. Volpert

**Affiliations:** ^1^Urology Department, Feinberg School of Medicine, Northwestern University, 303 East Chicago Avenue, Chicago, IL 60611, USA; ^2^Klinik für Urologie, Eberhard-Karls-Universität Tübingen, 72076 Tübingen, Germany

## Abstract

PPAR*γ* is a member of the ligand-activated nuclear receptor superfamily: its ligands act as insulin sensitizers and some are approved for the treatment of metabolic disorders in humans. PPAR*γ* has pleiotropic effects on survival and proliferation of multiple cell types, including cancer cells, and is now subject of intensive preclinical cancer research. Studies of the recent decade highlighted PPAR*γ* role as a potential modulator of angiogenesis in vitro and in vivo. These observations provide an additional facet to the PPAR*γ* image as potential anticancer drug. Currently PPAR*γ* is regarded as an important target for the therapies against angiogenesis-dependent pathological states including cancer and vascular complications of diabetes. Some of the studies, however, identify pro-angiogenic and tumor-promoting effects of PPAR*γ* and its ligands pointing out the need for further studies. Below, we summarize current knowledge of PPAR*γ* regulatory mechanisms and molecular targets, and discuss ways to maximize the beneficial activity of the PPAR*γ* agonists.

## 1. INTRODUCTION

**Figure 1:**
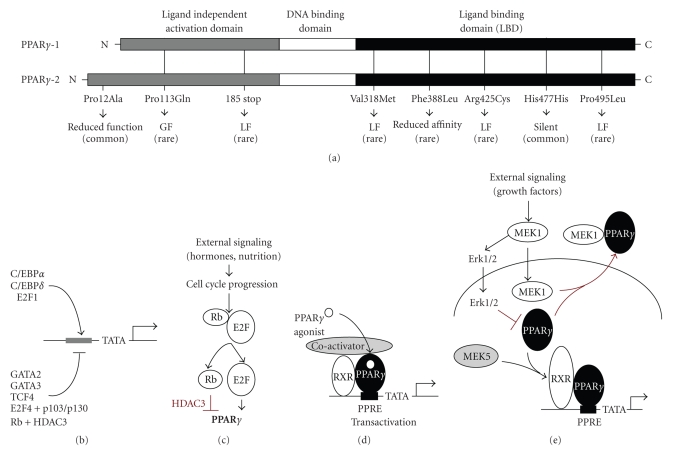
PPAR*γ* structure and regulation. (a) Schematic representation of the domain structure of the PPAR*γ*-1 and PPAR*γ*-2. The mutations associated with metabolic syndrome are indicated. LF: loss of function; GF: gain of function. (b) Positive and negative regulators of the PPAR*γ* gene transcription. (c) The regulation of PPAR*γ* levels by Rb and E2F. (d) The mechanism of ligand-dependent PPAR*γ* activation. (e) The regulation of PPAR*γ* activity by MEK and Erk kinases: MEK1 activates Erk-1/2, which phosphorylates PPAR*γ* and targets it to proteasomes; in addition, MEK1 binds PPAR*γ* in the nucleus and exports it to the cytoplasm. MEK5 can serve as coactivator for the PPAR*γ*.

PPARs are nuclear hormone receptors and targets for the compounds inducing peroxisome proliferation. The family encompasses three species, PPAR*α*, PPAR*β*/*δ*, and PPAR*γ*. PPAR*γ*, the best researched of the three, is presented by the two isoforms, *γ*1 and *γ*2 whereas PPAR*γ*2 contains 30 extra amino acids at the N-terminus due to initiation from the alternative transcription start (see Figure 1(a)). PPAR*γ*, a key player in adipocyte differentiation and glucose metabolism, is abundantly expressed in adipose tissues [1]. On the other hand, it is expressed in all the cells of the normal and pathological vascular beds, including endothelial cells (EC), macrophages (MΦ), and vascular smooth muscle cells (VSMCs), in a variety of tumor cells, and, at lower levels, in lymphatic tissue, intestinal epithelium, retina, and skeletal muscle [2]. PPAR*γ* is a potent modulator of the EC and VSMC function and inflammation: its effects on the tumor cells, tumor-associated MΦs (TAM), and tumor vasculature (EC and VSMCs) significantly attenuate tumor progression [3, 4], suggesting that PPAR*γ* ligands may become new convenient therapeutic modifiers targeting simultaneously tumors and their microenvironment [5]. Unfortunately, recent studies reveal the tumor-promoting and pro-angiogenic PPAR*γ* activities; while in most cases PPAR*γ* agonists attenuate tumor growth and angiogenesis, troglitazone (TGZ, a now rejected PPAR*γ* agonist) promotes hepatic carcinogenesis and liposarcomas. Moreover, some PPAR*γ* agonists promote the differentiation of the circulating endothelial progenitor cells (EPC) [6] and elicit angiogenesis in vivo [7]. In some instances, PPAR*γ* ligands increase the production of angiogenic stimuli, including VEGF or NO, by the EC or tumor cells [8]. Thus, the use of PPAR*γ* modulators to manage tumor progression is more complex than it appears at a glance and requires precise knowledge of the molecular events involved in their pro- and antitumorigenic actions. Below we summarize the current knowledge of PPAR*γ* effects and molecular mechanisms and delineate ways to augment PPAR*γ* anti-angiogenic and antitumor effects while minimizing its pro-angiogenic and tumor-promoting capacities.

## 2. PPAR*γ* AND ANGIOGENESIS

Angiogenesis is a complex process involving diverse cell types and controled by the pro- and anti-angiogenic factors produced by the ECs, VSMCs, and in vascular microenvironment by the stromal, tumor, and inflammatory cells. The balance between positive and negative angiogenesis regulators determines if the existing capillaries would expand, regress, or remain quiescent [9]. Active angiogenesis involves invasion, migration, and proliferation of the EC followed by the morphogenesis (assembly) of the neovessels. It is aided by the recruitment of the EPCs, which may constitute up to 50% of the cells in a neovessel [10]. The newly formed capillaries recruit vascular smooth muscle cells (VSMCs), which stabilize and render quiescent the newly formed capillaries: in thus stabilized mature vessels, the interactions between angiopoietin-1 (Ang-1) on the EC and Tie-2 receptor on the VSMCs generate signals that dampen EC sensitivity to the pro- and anti-angiogenic molecules [11]. Brown adipose tissue, a thermogenic organ in mammals responds to cold by increasing VEGF, thus creating permissive conditions for the fat expansion. Treatment of brown adipocytes with PPAR*γ* ligands reduces VEGF-C mRNA pointing to their anti-angiogenic potential [12]. Moreover, chimeric mice null for PPAR*γ* show gross defects in placental vascularization [13]. Natural and synthetic PPAR*γ* ligands block VEGF-driven angiogenesis in vivo, in matrigel implants, in rodent cornea, and choroid [14–16]. RGZ suppresses the growth and angiogenesis of the glioblastoma, Lewis lung carcinoma, liposarcoma, and rhabdomyosarcoma in mouse models [17], which is partly due to the PPAR*γ*-mediated apoptosis of the tumor EC and the repression of VEGF production by the tumor cells. Below, we elucidate the PPAR*γ* pleiotropic effects on angiogenesis and suggest optimization strategies.

## 3. PPAR*γ* REGULATORY MECHANISMS

PPAR*γ* can be regulated at expression level: PPAR*γ* gene is repressed by the GATA-2 and 3, TCF4 [18] (see Figure 1(b)), and transactivated by CAAT enhancer binding proteins (C/EBPs), predominantly C/EBP*α*, ADD1/SREBP1, and E2F1 (see Figure 1(b)) [19]. E2F proteins have dual effect on PPAR*γ* expression: during cell cycle progression, phospho-Rb releases E2F1 to activate PPAR*γ* promoter (see Figure 1(c)), however, E2F4, if bound to the p103 or p130 Rb, represses PPAR*γ* transcription [2, 18]. Moreover, hypo-phosphorylated Rb binds PPAR*γ* and recruits histone deacetylase (HDAC) 3 to the complexes, causing transcriptional repression (see Figure 1(c)) [19]. Multiple growth factors including platelet-derived growth factor (PDGF), basic fibroblast growth factor (bFGF), angiotensin II, tumor necrosis factor (TNF) *α*, interleukin (IL) 1*β*, and tumor-derived growth factor *β*(TGF-*β*) increase PPAR*γ* expression by the vascular smooth muscle cells (VSMCs), via Egr-1. In contrast, AP-1 aided by Smad3/4 represses PPAR*γ* promoter activity [20]. Mitotic, stress, and inflammatory signals cause PPAR*γ* degradation via phosphorylation on Ser84 of the mouse PPAR*γ* (Ser112 of the human molecule) in a consensus MAPK target motif PXSPP [21] by ERKs, JNKs, and p38, which leads to ubiquitination and proteasomal clearance [22]. Ser to Ala PPAR*γ* mutant shows increased transcriptional activity, similar effect is caused by coexpression of a phosphoprotein phosphatase [21]. In human PPAR*γ*, substitution of proline to glutamine at position 115 results in constitutive activation by blocking MAPK phosphorylation at position 114: patients with such mutation display extreme obesity [23]. Likewise, increased phosphorylation on Ser112 in Dok-1 null mice caused lean phenotype, which is lost in mice expressing phosphorylation-defective PPAR*γ* [24]. The effect of PPAR*γ* on angiogenesis remains to be determined.

The next regulatory step involves cofactor recruitment: upon ligand binding, PPAR*γ* forms heterodimers with the retinoic acid X receptor (RXR), and occupies twin PPAR response elements AAGGTCAnAAGGTCA (PPRE); binding of the RXR ligands further increases transcriptional activity of the PPAR*γ*/RXR dimers (see Figure 1(d)). Coactivators including SRC1, CBP/p300, pCAF/GCN, and PGC bind PPAR*γ*/RXR complexes in a ligand-dependent manner [19]; PGC-1*α* has recently been linked to HIF-independent induction of vascular endothelial growth factor (VEGF) and angiogenesis [25]. PPAR*γ* activity can be suppressed due to phosphorylation, which results in nuclear export, both executed by MEK-1 (see Figure 1(e)) [26]. In contrast, MEK-5 acts as PPAR*γ* coactivator (see Figure 1(e)) [27].

**Figure 2:**
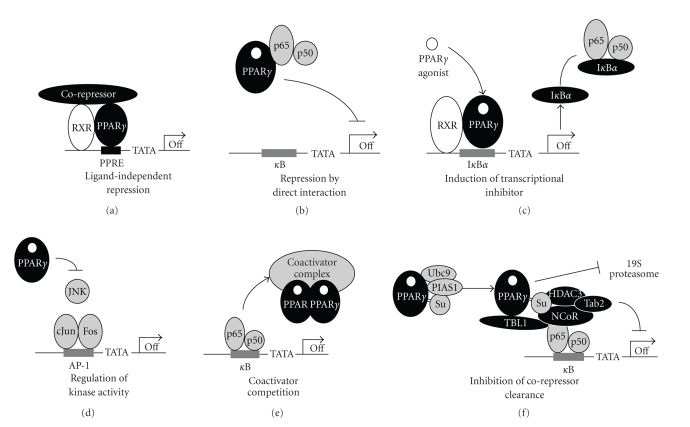
Mechanisms of transrepression by PPAR*γ*. (a) Ligand-independent repression: preferential recruitment of corepressors in the absence of agonists. (b) Direct binding and sequestration of transcription factors on example of NF*κ*B. (c) Activation of genes encoding inhibitors of transcription factor (e.g., NF*κ*B inhibitor, I*κ*B*α*). (d) Direct binding and inactivation of kinases, which activate transcription factors (e.g., the blockade of JNK activation of cJun). (e) Competitive binding of the coactivator complex. (f) The blockade of corepressor clearance: sumoylated PPAR*γ* stabilizes corepressor complexes (NCoR, Tab2, and TBL1) on the promoter and facilitates the recruitment of HDAC3. In the absence of sumoylation, NCoR, Tab2, and TBL1 are subject to ubiquitination and proteasomal clearance.

In addition to its activator function (see Figure 1(d)), PPAR*γ* represses transcription of select genes. PPAR*γ* transrepression of AP-1, nuclear factor of the activated T-cells (NFAT), NF*κ*B, and STAT-1 is well documented [19, 28]. Typical PPAR*γ* corepressors SMRT and NCoR corecruit HDAC3, transducin beta-like protein-1 (TBL-1) and TBL-1-related protein 1 (TBLR1) [29]. The repression can be ligand-independent, with PPAR/RXR dimers forming repressor complexes in the absence of the ligands (see Figure 2(a)). Ligand-dependent repression may occur by direct interaction with target transcription factors (see Figure 2(b)), modulation of the transcriptional regulators (see Figures 2(c) and 2(d)), by coactivator sequestration (see Figure 2(e)), or the blockade of corepressor clearance (see Figure 2(f)). The latter requires PPAR*γ* sumoylation, which keeps HDAC3 associated with repressor complexes and prevents proteasomal clearance of their components [19]. NCoR complexes interact with a limited subset of promoters, which explains gene-specific repression by PPAR*γ*.

## 4. LIGANDS

PPAR*γ* ligands encompass wide range of structurally diverse compounds, natural and synthetic. Natural ones include long chain polyunsaturated fatty acids and derivatives (eicosanoids, prostaglandins, like 15-deoxy-Δ^12,14^-prostaglandin J_2_ (15D-PGJ_2_)) and nitrolinoleic acids. Synthetic ones include thiazolinediones (TZDs, or glitazones), of which rosiglitazone (RGZ) and pioglitazone (PGZ) are marketed for the treatment of type 2 diabetes and tyrosine-based derivatives (glitazars) including tesaglitazar and farglitazar, the dual agonists of PPAR*α* and PPAR*γ* [30]. Although their ability to alleviate insulin resistance, vascular complications, and angiogenesis is well documented, the adverse effects include hepatotoxicity, renal toxicity, weight gain, and fluid retention [30], all of which complicate the long-term use. Thus further work is required to develop PPAR*γ* ligands into safe and efficacious treatment for diabetes, cancer, and angiogenesis-related disease. Selective PPAR*γ* modulators (SPPARMs) represent one way to overcome this problem: they are designed to retain the desired PPAR*γ* properties, while minimizing adverse side effects. SPPARMs can be categorized as tightly binding partial agonists (GW0072) or weakly binding full agonists of PPAR*γ* (MCC-555/netoglitazone, NC-2100) [31].

## 5. ANTI-ANGIOGENIC EFFECTS OF PPAR*γ* IN DIVERSE CELL TYPES: ENDOTHELIAL-SPECIFIC EVENTS

Human micro- and macrovascular endothelial cells (EC) express PPAR*γ* [32]. PPAR*γ* activation by the natural (15D-PGJ_2_) or synthetic ligands (TGZ, RGZ, ciglitazone, and pioglitazone) potently inhibits in vitro proliferation and morphogenesis by EC of diverse tissue origin [33]. 15D-PGJ_2_ and ciglitazone (CGZ) also induce EC apoptosis through PPAR*γ*-dependent pathway. The PPAR*γ* involvement is supported by (1) nuclear translocation, (2) increased transcriptional activity, (3) attenuation of the EC apoptosis by the decoy PPRE oligonucleotide, and (4) increased background apoptosis in PPAR*γ* overexpressing EC, further enhanced by the ligand exposure [15]. PPAR*γ* activation interferes with EC migration: TZDs block EC chemotaxis up the VEGF or leptin gradients, by blocking PI3K/Akt and Erk1/2 signaling [34–37]. In both cases, PPAR*γ*/SREBP1 complex drives the transcription of PTEN tumor suppressor, which opposes the induction of Akt [38], see Figure 4(a).

PPAR*γ* ligands hamper the response of the vascular EC to VEGF by lowering VEGFR1 (Flt-1) and VEGFR2 (KDR). The regulation of VEGFR2 is biphasic: in the absence of the ligands, PPAR*γ* enhances Sp1/Sp3 binding to the promoter and opposes it if ligands are present [39]. VEGFR2 decrease also reduces EC survival under stress or in the presence of anti-angiogenic factors, see Figure 4(a).

PPAR*γ* induction decreases UPA and increases PAI-1 expression by the EC, thus lowering their ability to invade surrounding tissues [14, 16]. In the brain microvasculature, PPAR*γ* stimulation dampens the activation of RhoA and Rac1 GTPases critical for the cell adhesion and migration [40], see Figure 4(a).

Proapoptotic PPAR*γ* effects in the EC can be mediated by p53 [41–43] or by the opening of Maxi-K channel (Ca2^+^ activated K^+^ channel) whereas the protective Bcl-2 levels plummet and apoptotic Bax increases. In addition, increased eNos production causes elevated NO, which, in contrast with its usual protective effect contributes to EC death [44]. Downmodulation of the thioredoxin (Trx-1) by PPAR*γ* via vitamin D3 upregulated protein (VDUP-1) also contributes to the EC killing, likely via formation of inactive PTEN/Trx-1 complexes [45]. PPAR*γ* also ameliorates EC activation by glucose via the induction of diacylglycerol kinase (DGK), the reduction of diacylglycerol, which attenuates PKC activity and decreases angiogenesis [46]. Importantly, PPAR*γ* activation enhances surface CD36, a lipid scavenger receptor, which transmits the anti-angiogenic signal of thrombospondin-1 (TSP1) [47] a potent endogenous inhibitor of angiogenesis, see Figure 4(a).

PPAR*γ* produces complex effect on the endothelial progenitor cells (EPC): RGZ enhances the expression of the endothelial markers CD31 and VEGFR2 on the circulating EPCs, however VE-cadherin and CD146 remain low; increased uptake of oxidized lipids suggests elevated CD36, which increases the sensitivity to TSP1. EPCs from the diabetic patients treated with RGZ display better adherence to fibronectin than those from untreated diabetics and normal donors [6]. This is consistent with reduced oxidative stress and improved re-endothelialization by the EPCs from diabetic patients in RGZ-treated mice [48]. EPCs from the RGZ-treated diabetics migrate more vigorously than those from untreated subjects, but similarly to the EPC from untreated normal donors [6] suggesting that RGZ rather normalizes than increases the EPCs migratory potential. PGZ effect on cultured EPCs is twofold: it enhances the expression of endothelial markers at a lower dose (1 *μ*m) and reduces it at higher (10 *μ*m) concentration. PGZ also stimulates the expression of TGF*β* and TGF*β* receptor [49], and thus initiates EPC conversion to the VSMC phenotype [50]: increased VSMC presence may stabilize the neovasculature and thus reduce angiogenesis. This may explain why PPAR*γ* agonists ameliorate glomerulonephritis in mouse model without increase in EPC homing [51].

## 6. IN VASCULAR SMOOTH MUSCLE CELLS

Genetic variations associated with atherosclerosis point to PPAR*γ* role in associated metabolic and vascular events [52]. In atherosclerotic lesions, PPAR*γ* promotes vascular repair and re-endothelialization, while suppressing neointima formation. PPAR*γ* attenuates vasoconstrictive remodeling by blocking NADPH oxidases [53] and inhibits VSMCs proliferative and migratory responses to multiple cytokines and growth factors including PDGF-BB, bFGF, thrombin, insulin, and angiotensin II (AngII). PPAR*γ* interferes with VSMC proliferation and survival by blocking the downstream targets of ERK1/2 and PI3K/Akt, SHIP2 and two important regulators of mRNA translation, p70S6 kinase and 4-EBP translation initiation inhibitor [54]. In addition, PPAR*γ* activation enhances the expression of Shp-2 phosphatase, which dephosphorylates/inactivates Vav, a guanidine exchange factor for RhoA, impairs the activation of Rho-associated kinase (ROCK), and suppresses VSMC proliferation and migration [55]. PPAR*γ* inhibits VSMC migration but not the attachment and motility components of the migratory response: the inhibition of PDGF-BB driven VSMC migration is due to the transcriptional repression of Ets-1, which, in turn, drives MMP-9 and invasion [56], see Figure 4(b).

PPAR*γ* activation causes VSMC growth arrest via multiple pathways: (1) by suppressing proteasomal degradation of the p27/Kip; (2) via transrepression of the E2F target, minichromosome maintenance protein, MCM7, which blocks replication [2]; (3) by blocking Ets-1 dependent transactivation of telomerase promoter [57]. PPAR*γ* and its agonists potently induce VSMC apoptosis (1) through direct upregulation of GADD45 and p53 via an Oct-1 dependent mechanism (PPRE are identified in GADD45 and p53 promoters) [58, 59]; (2) by inducing the TFG-*β*/ALK/Smad pathway, subsequent Bcl-2 repression, and Smad-dependent induction of GADD45 [60]; (3) through transcriptional upregulation of the interferon regulatory factor-1 (IRF-1), a proapoptotic, antiproliferative transcription factor [61], see Figure 4(b).

All PPAR*γ*-dependent changes in VSMC behavior can contribute to its anti-angiogenic function: decreased VSMC migration, and proliferation, plus increased apoptosis restrict VSMC incorporation in the vasculature and therefore the stability of neovessels. Moreover, ECs of the immature, VSMC-poor vessels are vulnerable to the apoptotic signals by angiogenesis inhibitors, see Figure 4(b).

## 7. ANTI-INFLAMMATORY EFFECTS

PPAR*γ* affects inflammation directly, by driving CD36-dependent apoptosis in MΦs [62, 63], or indirectly, by reducing VCAM-1 expression by the ECs and thus blocking transendothelial migration (TEM) of monocytes and MΦs during chronic inflammation typical for diabetes and cancer. In contrast, E-selectin, a mediator of the acute immune response, is not altered by PPAR*γ* [64]. Statins increase anti-inflammatory Cox-2 in MΦs, which, in turn, increases endogenous 15D-PGJ_2_, activates PPAR*γ*, and upregulates its downstream target, CD36 [65]. In addition, PPAR*γ* ligands cause NF*κ*B transrepression, thus reducing the production of inflammatory cytokines (IL-8, IL-6, MCP-1, and CX3CL1-1) by MΦs, and thus disrupting paracrine loop that attracts tumor-associated MΦs (TAM) and thus stimulates angiogenesis and tumor growth [66], see Figure 4(c).

## 8. IN TUMOR CELLS AND STROMA

PPAR*γ* is expressed in human carcinomas of the breast, colon, esophagus, liver, lung, pancreas prostate, stomach, and thyroid, also in neuroblastoma, astrocytoma, and glioma: in all of these PPAR*γ* ligands repress or delay xenograft growth in mouse models [67].

**Figure 3:**
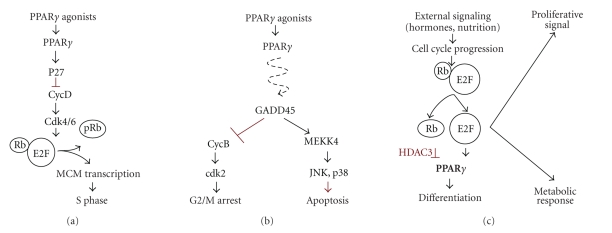
PPAR*γ* effects in cancer cells. (a) The induction of Cdk inhibitor, p27 causes growth arrest due to reduced MCM7 activity and subsequent blockade of replication. (b) The induction of GADD45 impairs Cyclin B and causes G2M growth arrest. In addition, the activation of JNK and p38 kinases via MEKK4 initiates cell death by apoptosis. (c) PPAR*γ* activation by hormones and nutrition in normal cells and by agonists in cancer cells may activate the differentiation programs.

**Figure 4:**
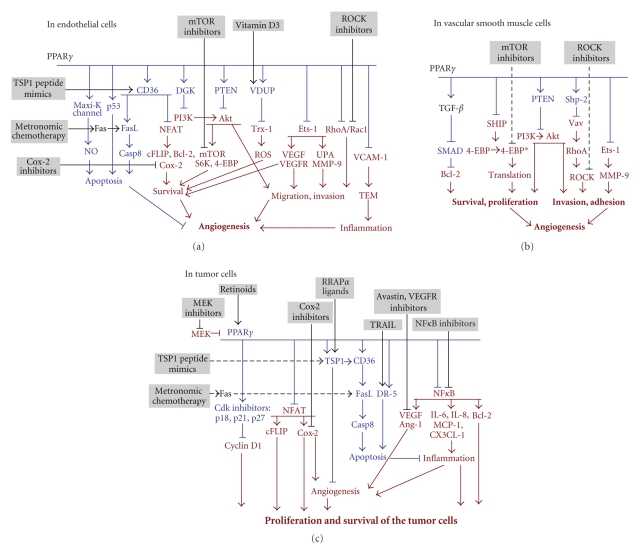
PPAR*γ* effects on the endothelial, pericytic, tumor and immune cells in the tumor microenvironment: the consequences of angiogenesis and possible ways to augment antitumor actions. Pro-angiogenic and tumor-promoting events are shown in red. The opposing effects are in blue. The proposed drugs are shown in black. (a) Summary of the PPAR*γ* molecular effects in the endothelial cells. TEM, transendothelial migration. (b) PPAR*γ* molecular effects on the VSMCs. (c) The effects on macrophages and tumor cells.

PPAR*γ* ligands affect tumor cells in several ways: they reduce proliferation, enhance apoptosis, and modulate angiogenic phenotype of the tumor cells. PPAR*γ* targets cyclin D1 via the inhibitors of cyclin-dependent kinases (Cdk), p18, p21, and p27, causing a decline in Rb phosphorylation [1] and arresting cells in G1 phase: PPAR*γ* acts via p21 and p27 in pancreatic cancer and via p18 in hepatoma (see Figure 3(a)). On the other hand, glitazones repress the production of Cdk2, 4 and 6 in carcinomas of the bladder, breast, lung, and pancreas via GADD45 [67] (see Figure 3(b)). PPAR*γ* activation also restores PTEN expression in tumor cells and thus blocks PI3K/Akt axis [38], it can also initiate a negative feedback loop, which consists of calcineurin phosphatase, nuclear factor of the activated T-cells (NFAT), and down syndrome critical region 1 (DSCR1), which inhibits calcineurin and blocks NFAT activity necessary for proliferation and survival (see Figure 3(c)) [68], see Figure 4(c).

PPAR*γ* induction also causes tumor cell apoptosis by downmodulating prosurvival proteins cFLIP and Bcl-2, while increasing proapoptotic Bax and BAD, as occurs in glioblastoma [69] or by the interference with the PI3K/Akt signaling [38]. Conversely, PPAR*γ* often augments the expression of TNF-related apoptosis inducing ligand (TRAIL), which selectively eliminates cancer cells [70], see Figure 4(c).

In some cases, PPAR*γ* activation induces tumor cell differentiation (e.g., liposarcoma, breast and pancreatic cancer, neuroblastoma, glioma, bladder carcinoma, and lung carcinoma). The differentiation is evidenced by the increase of the general markers of differentiated state, such as E-cadherin, and downregulation of the specific markers of progenitor lineages, also by morphology changes consistent with differentiated state (see Figure 3(d)) [1, 67].

Finally, treatment with the PPAR*γ* ligands frequently downregulates the expression of pro-angiogenic factors VEGF [17], IL-8 [71], Ang-1 [72], and Cox-2 [73] and thus suspends tumor angiogenesis. Moreover, mice null for PPAR*γ* show impaired tumorgenesis, due to the dramatic increase in TSP-1 [5], see Figure 4(c).

## 9. PPAR*γ* PRO-ANGIOGENIC/TUMORIGENIC EFFECTS

In contrast to the majority of findings, a recent study suggests that PPAR*γ* ligands may have pro-angiogenic properties both in vitro [74], in an endothelial/interstitial cell coculture assay, and in a murine corneal angiogenesis model in vivo [74]. The magnitude of the angiogenic response caused by PPAR*γ* ligands has not been compared to the angiogenesis elicited by typical stimuli (VEGF, bFGF); also, the contradiction between these results and previous studies has not yet been addressed.

PPAR*γ* pro-angiogenic effects are associated with the induction of VEGF and increased phosphorylation of eNOS and AKT [7, 75], which cause elevated VEGF production in human and rodent VSMCs, MΦs and tumor cells [76–79], VEGF and VEGFR levels in the ECs and myofibroblasts [80]. Although PPAR*γ* ligands inhibit xenografted human tumors [1, 33], in one study using mouse model of colon cancer (APC/Min) PPAR*γ* ligands increased the number of precancerous polyps, tumor frequency and size [81]. However, in two other models, APC-deficient HT-29 xenografts and azoxymetane-induced tumors PPAR*γ* ligands suppress tumor growth and angiogenesis [82, 83]. Of the multiple small-scale clinical trials using PPAR*γ* ligands for cancer treatment, only two showed promising results: in an early study TGZ caused prolonged PSA stabilization in prostate cancer patients [84], while PGZ combined with low-dose chemotherapy and rofexoxib produced moderate improvement in the patients with high-grade glioma [85]. In contrast, patients with breast, colon, and thyroid cancers showed no significant response [86–88]. Thus, the use of PPAR*γ* ligands in clinical practice obviously requires optimization, and the answers may come from the use of combination or complementation treatments.

## 10. PPAR*γ* LIGANDS IN COMBINATION TREATMENTS: CAN WE AUGMENT THE BENEFICIAL EFFECTS?

The information above narrows down the list of PPAR*γ* targets critical for its anti-angiogenic and antitumor effects (see Figure 4(a)). PPAR*γ* reverses angiogenic functions in the ECs by blocking the expression of VEGF-A and its receptor, VEGFR2 by blocking Ets-1 transcription factor, and by dampening the prosurvival PI3K/Akt cascade, likely via PTEN induction. It also deactivates RhoA/Rac1 small GTPases which enable EC migration. NFAT deactivation lowers the levels of the apoptosis inhibitors, cFLIP and Bcl-2, and critical invasion molecules UPA and MMP 9. In addition, PPAR*γ* promotes the following proapoptotic events: it elevates expression of the proapoptotic CD36 and TSP1 receptor-ligand duo; increases p53 stability; opens of the Maxi-K channel to upregulate nitric oxide (NO), which, paradoxically, causes apoptosis. In addition, PPAR*γ* suppresses Trx-1 and ROS levels by upregulating VDUP-1, a vitamin D3 target. Finally, PPAR*γ* ligands block protein synthesis via 4-eBP and p70S6 kinase, both the targets of mTOR pathway.

In the VSMC, PPAR*γ* represses the activation of prosurvival Erk-1 and PI3K/Akt and SHIP thus sustaining the unphosphorylated, active state of 4-EPB, a negative regulator of translation. It also enhances the activity of Shp-2 phosphatase, which blocks Vav, the trigger of RhoA/ROCK pathway necessary for survival and migration; PPAR*γ* also interferes with VSMC Bcl-2 expression by enhancing TGF*β*/Smad2 and disrupts MMP-9 production by blocking Ets-1 (see Figure 4(b)).

In MΦs and tumor cells, PPAR*γ* through transrepression of NF*κ*B and NFAT lowers the production of multiple growth factors and inflammatory cytokines including VEGF, Ang-1, cyclo-oxygenase (Cox) 2, IL-6, IL-8, MCP-1, and CX3CL-1. PPARg also enhances the production of thrombospondin (TSP) 1: therefore angiogenic balance tips in favor of vascular quiescence. In addition, PPAR*γ* lowers the resistance of tumor cells and tumor-associated MΦs (TAM) to stress and apoptotic stimuli by blocking cyclin D1 via cdk inhibitors p18, p21, p27, by repressing antiapoptotic Bcl-2 and FLIP, by upregulating proapoptotic CD36 in MΦs, and Bax and BAD in tumor cells (see Figure 4).

This comprehensive list of PPAR*γ* targets and interacting proteins can be used for intelligent design of the optimal combination therapies based on PPAR*γ* ligands to achieve the best anti-angiogenic and anticancer activity. For example, it stands to reason to expect that EC apoptosis caused by PPAR*γ* can be augmented by supplying CD36 ligand, TSP1 or its peptide mimics, such as ABT-510 [89]. Indeed, PPAR*γ* ligands 15PG-E2, TGZ and RGZ, and TSP1 anti-angiogenic peptide ABT-510 synergistically block angiogenesis and curtail the growth of lung and bladder carcinoma xenografts, by initiating CD36-dependent apoptotic events in remodeling tumor endothelium [47]. Furthermore, TSP1 expression is enhanced by the low-dose metronomic chemotherapy, including cytoxan, docetaxel, and 5-fluorouracil [90–92]. Thus cytoxan, docetaxel, and 5-fluorouracil are likely to potentiate the PPAR*γ* anti-angiogenic effects in EC and to reduce tumor-associated inflammation responses by killing TAMs. This is supported by the fact that 15D-PGE_2_ enhances antitumor activity of docetaxel against lung carcinoma cell lines [93]. In addition, metronomic chemotherapy enhances the expression of Fas, a critical apoptosis mediator induced by the TSP1/CD36 interaction and thus potentiates the activity of TSP1 derivatives, such as ABT-510 [94, 95]. Hence, combined use of PPAR*γ* ligands and metronomic regimens of chemotherapy agents is likely to be more effective than individual treatments.

PPAR*γ* blockade of the EC and VSMC migration involves the inhibition of RhoA/ROCK signaling [40, 65], which makes ROCK inhibitors likely candidates for the use in combination with PPAR*γ* ligands. This is doubly important, since ROCK activates Myc pathway and thus abolishes TSP1 expression by the tumor cells [96]. ROCK inhibitors show strong toxic effects at therapeutic doses, thus their clinical use is problematic. However, combined use with PPAR*γ* ligands may allow to lower their effective concentration and therefore limit drug-induced toxicity.

Since PPAR*α* strongly increases TSP1 production, combined use of PPAR*α* and PPAR*γ* agonists or the use of dual PPAR*α*/*γ* ligands may present an advantage. Interestingly, TZD18, a novel PPAR*α*/*γ* dual agonist induces apoptosis of glioma cells with high efficiency [97]. Unfortunately, glitazars have carcinogenic activity of their own [98].

PPAR*γ* ligands sensitize leukemic, lung and endothelial cells to the TRAIL-induced apoptosis by enhancing DR5 expression [99, 100] pointing to possible synergy between PPAR*γ* agonists and TRAIL therapies.

The inhibition of VEGFR2 expression by vascular endothelium, which contributes to the antiangiogenesis by the PPAR*γ*, could be assisted by VEGF sequestering agents, such as Avastin, or by the inhibitors VEGF RTK activity, such as sunitinib, sorafenib or VEGF decoy receptor. This hypothesis is yet to be tested.

The downstream target of the PI3K/Akt pathway, which is blocked by PPAR*γ* via PTEN activation, is tuberous sclerosis tumor suppressor complex, which, when phosphorylated by Akt, allows the activation of mammalian target of rapamycin (mTOR) kinase, protein synthesis, and cell survival [101]. On the other hand, PPAR*γ* ligands interfere with translation by augmenting the activity of 4-EBP and blocking S6 kinase [102]. Thus PPAR*γ* disrupts mTOR regulation of protein synthesis at two distinct steps. Moreover, the blockade of mTOR pathway is likely to suppress VEGF in all cell types in the tumor microenvironment [103]. Hence, mTOR inhibitors such as tacrolimus are likely to complement the anti-angiogenic and antitumor activity of PPAR*γ* agonists. Cyclic AMP analogs, which block mTOR activity via AMPK1 pathway [101], may also contribute to the PPAR*γ* beneficial effects: this is particularly important, since cAMP analogs are capable of increasing PPAR*γ* activity (Schulze-Hoepfner and Volpert, unpublished observations). The fact that amino acid deprivation, the main off switch for the mTOR, enhances PPAR*γ* proapoptotic effects in tumor cells [104] lends further support to this hypothesis.

PPAR*γ* transrepression of NF*κ*B and NFAT signaling leads to the inhibition of multiple angiogenic stimuli, including interleukins 6 and 8, MCP-1 and CX3CL-1, as well as protective Ang-1 and proinflammatory Cox-2. This PPAR*γ* function suggests a wide range of possible treatment combinations with NF*κ*B inhibitors, including synthetic inhibitors of IKK kinases [105] or naturally occurring plant substances, like curcumin [106]. On the other hand, the inhibition of Cox-2 with highly selective agents, like celexoxib, has direct anti-angiogenic tumor-preventing effects [107] and is quite likely to contribute to the PPAR*γ* antitumor and anti-angiogenic activities, especially in the light of potentiating effect of celexoxib on docetaxel treatment [108] and beneficial effects of PGZ combined with rofexoxib and low-dose chemotherapy [85].

PPAR*γ* activity is opposed by MEK kinases: thus MEK inhibitors are likely to improve the efficacy of PPAR*γ* ligands: indeed, MEK-1 inhibitor, PD98059, improves CGZ antitumor effect in colon cancer xenografts [109]. PPAR*γ* activity is also augmented by RXR ligands: 9-cis retinoic acid (RA) enhances PPAR*γ*-induced differentiation and gene expression. In colon cancer, PPAR*γ* and RXR ligands induce differentiation and apoptosis more potently than each individual compound [110, 111]. Nine-cis retinoic acid partially overcomes RXR phosphorylation, which reduces PPAR*γ*/RXR dimerization and opposes PPAR*γ* activity: MEK-1 inhibitors improve the combined effect of CGZ and 9-cis RA [109]. Finally, HDAC inhibitor, trichostatin A, potentiates the effects of phenolfibrate on the differentiation and attenuation of stemness of the lung adenocarcinoma cells [112]. While combining PPAR*γ* agonists with other drugs, particular attention should be paid to the agonist dosage: studies of PPAR*γ* effects metabolic syndrome demonstrate that overactive and hypoactive mutants cause similar metabolic consequences and suggest the use of SPPARMs versus full agonists [113].

The list of agents with the potential to enhance the antitumor and anti-angiogenic effects of PPAR*γ* ligands is not limited by the examples above, however we hope that it provides a convincing example of rational design of the complementation therapies, based on the knowledge of molecular mediators of a given agent. The examples, which demonstrate the improved efficacy of predicted combinations, provide an impetus for the evaluation of the combinations, which have not yet been tested.

## ABBREVIATIONS

ADD1: Adducin 1
AMPK1: Adenosine Monophosphate Protein Kinase
Ang-1: Angiopoietin-1
APC: Adenomatous Polyposis Coli
Bcl: B-cell Leukemia
bFGF: Basic Fibroblast Growth factor
C/EBPs: CAAT enhancer binding proteins
CBP: CREB binding Protein
Cdk: Cyclin-Dependent Kinases
cFLIP: FLICE Inhibitory Protein, a caspase-8 inhibitor
CGZ: Ciglitazone
Cox: Cyclooxygenase
DGK: Diacylglycerol Kinase
15D-PGJ_2_: 15-deoxy-Δ^12,14^-prostaglandin J_2_
DSCR1: Down Syndrome Critical Region 1
EC: Endothelial Cells
Egr: Early Growth Response
EPC: Endothelial Progenitor Cells
ERKs: Extracellular Signal-Regulated Kinase
GATA: GATA-binding transcription factor
HDAC: Histone deacetylase
HIF: Hypoxia Inducible factor
IKK: Inhibitor of Kappa Beta Kinase
IL: Interleukin
MAPK: Mitogen-Activated Protein Kinase
MCM7: Minichromosome Maintenance Protein
MCP: Monocyte Chemotactic Protein-1
MMP: Matrix Metalloproteinase
mTOR: Mammalian Target of Rapamycin
NCoR: Nuclear Co-Repressor
NADPH: Nicotinamide Dinucleotide Phosphate
NFAT: Nuclear Factor of the Activated T-cells
NF*κ*B: Nuclear Factor Kappa Beta
JNKs: Jun N-terminal Kinase
MΦ: Macrophages
PPAR: Peroxisome Proliferator Activated Receptor
PAI-1: Plasminogen Activator Inhibitor
PDGF-BB: Plateled-Derived Growth Factor
PI3K: Phosphatidil Inositol-3 Kinase
PGZ: Pioglitazone
PPRE: PPARg Response Element
PTEN: Phosphatase and Tensin Analog
Rb: Retinobalsoma
ROCK: Rho-associated kinase
RXR: Retinoic Acid X receptor
STAT: Signal Transducers and Activators of Transcription
SMRT: Silencing Mediator of Retinoic Acid and Thyroid Hormone Receptor
SPPARMs: Selective PPAR*γ* modulators
SREBP: Serum response Element Binding Protein
TAM: Tumor-associated Macrophages
TBL-1: Transducin Beta-Like Protein-1
TCF: T-cell factor
TNF: Tumor Necrosis Factor
TGF-*β*: Tumor-Derived Growth Factor *β*
TGZ: Troglitazone
TRAIL: TNF-related apoptosis inducing ligand
Trx: Thioredoxin
TSP1: Thrombospondin-1
TZDs: Thiazoliediones
UPA: Urokinase Plasminogen Activator
VDUP: Vitamin D3 Upregulated Protein
VEGF: Vascular Endothelial Growth Factor
VSMC: Vascular Smooth Muscle Cells
VCAM: Vascular Cell Adhesion Molecule-1.
